# (*RS*)-3-Acetyl-2-methyl-4-(3-nitro­phen­yl)-1,4,5,6,7,8-hexa­hydro­quinolin-5-one

**DOI:** 10.1107/S160053680903832X

**Published:** 2009-09-26

**Authors:** Dong’e Wang, Yu-zhou Wang, Muhtar Turhong

**Affiliations:** a Department of Chemistry, Kashgar Teachers’ College, Kashgar 844000, People’s Republic of China

## Abstract

In the title compound, C_18_H_18_N_2_O_4_, the nitro group, a methyl group, the acetyl group and some atoms of the dihydro­quinolinone group are disordered over two sites with the ratio of occupancies fixed at 0.57:0.43. The relationship between the major and minor components of disorder is that of diastereomers. In the crystal structure, inter­molecular N—H⋯O, weak C—H⋯O and C—H⋯π inter­actions link the mol­ecules into two-dimensional layers running parallel to the (010) plane.

## Related literature

For the biological importance of polyhydro­quinoline derivatives, see: Ko & Yao (2006 ▶). For bond-length data, see: Allen *et al.* (1987 ▶). 
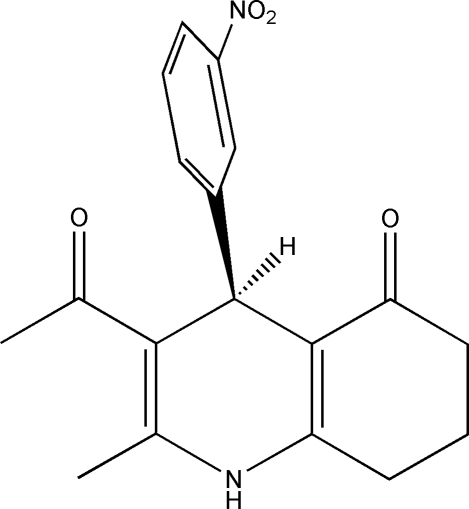


## Experimental

### 

#### Crystal data

C_18_H_18_N_2_O_4_
*M* *_r_* = 326.34
Monoclinic, 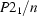

*a* = 8.5368 (5) Å
*b* = 17.0307 (6) Å
*c* = 11.4759 (5) Å
β = 106.143 (1)°
*V* = 1602.67 (13) Å^3^
*Z* = 4
Mo *K*α radiation
μ = 0.10 mm^−1^
*T* = 298 K
0.20 × 0.10 × 0.10 mm

#### Data collection

Bruker SMART APEX CCD area-detector diffractometer
Absorption correction: multi-scan (*SADABS*; Sheldrick, 1996 ▶) *T*_min_ = 0.971, *T*_max_ = 0.990
9050 measured reflections
3151 independent reflections
1611 reflections with *I* > 2σ(*I*)
*R*_int_ = 0.094

#### Refinement

*R*[*F* ^2^ > 2σ(*F* ^2^)] = 0.061
*wR*(*F* ^2^) = 0.149
*S* = 0.90
3151 reflections
325 parameters
12 restraints
H atoms treated by a mixture of independent and constrained refinement
Δρ_max_ = 0.34 e Å^−3^
Δρ_min_ = −0.28 e Å^−3^

### 

Data collection: *SMART* (Bruker, 2001 ▶); cell refinement: *SAINT-Plus* (Bruker, 2001 ▶); data reduction: *SAINT-Plus*; program(s) used to solve structure: *SHELXS97* (Sheldrick, 2008 ▶); program(s) used to refine structure: *SHELXL97* (Sheldrick, 2008 ▶); molecular graphics: *PLATON* (Spek, 2009 ▶); software used to prepare material for publication: *PLATON*.

## Supplementary Material

Crystal structure: contains datablocks global, I. DOI: 10.1107/S160053680903832X/lh2906sup1.cif

Structure factors: contains datablocks I. DOI: 10.1107/S160053680903832X/lh2906Isup2.hkl

Additional supplementary materials:  crystallographic information; 3D view; checkCIF report

## Figures and Tables

**Table 1:** Hydrogen-bond geometry (Å, °)

*D*—H⋯*A*	*D*—H	H⋯*A*	*D*⋯*A*	*D*—H⋯*A*
N2—H2*A*⋯O3^i^	0.868 (10)	2.063 (13)	2.927 (8)	173 (2)
C6—H6⋯O4^i^	0.93	2.56	3.309 (16)	138
C18—H18*A*⋯O1^i^	0.96	2.46	3.239 (18)	138
C18—H18*B*⋯O3^i^	0.96	2.57	3.422 (15)	148
C11—H11*A*⋯O1^ii^	0.97	2.55	3.290 (16)	133
C12—H12*A*⋯*Cg*1^iii^	0.97	2.76	3.71 (1)	166

Symmetry codes: (i) 
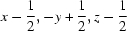
; (ii) 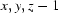; (iii) 
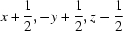
. *Cg*1 is the centroid of the C1–C6 ring.

**Fig. 1.**
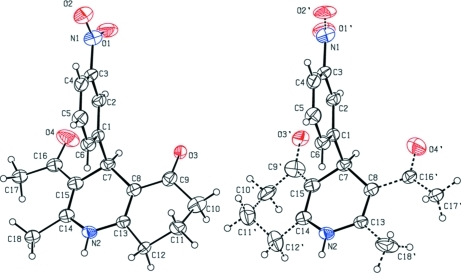
The title molecule with the atom-numbering scheme, showing both the major (left) and minor (right) components. The displacement ellipsoids are drawn at the 30% probability level.

**Fig. 2.**
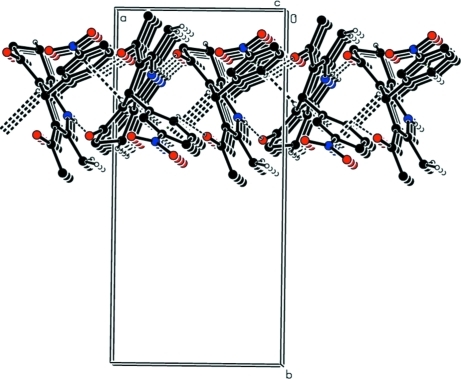
Part of the crystal packing, showing the two-dimensional (010) layer. The weak hydrogen bonds and C—H···π interactions are shown as dashed lines. For the sake of clarity, the H atoms and disordered atoms not involved in the hydrogen-bonds pattern have been omitted.

## supplementary crystallographic information

### Comment

Many polyhydroquinoline derivatives have been synthesized because of their biological importance (Ko & and Yao). In this paper, we report the crystal structure of the title compound, (I).

In the title molecule (Fig. 1), the nitro group, C18 methyl group, the acetyl group and some atoms of the dihydroquinolin-one (i.e. atoms C9-C12/C16-C18/O3/O4) are disordered over two positions with final site occupancies of 0.57:0.43 for the major and minor components, respectively. The bond lengths and angles are within normal ranges (Allen *et al.*, 1987).

In the crystal structure, the molecules are first linked by one N2—H2A···O3 (-1/2+x,1/2-y,-1/2+z) hydrogen bond  into a one-dimensional  chain running parallel to the [101] direction (Table 1 and Fig. 2). These adjacent [101] chains are joined together by C11–H11A···O1 (x, y, -1+z) and C-H···π (C12···Cg1 =3.71 (1)Å, Cg1 is the centroid defined by atoms C1-C6 at (1/2+x,1/2-y,-1/2+z)) interactions, forming a two-dimensional layer structure running parallel to the (010) plane.

### Experimental

The title compound was synthesized according to a literature procedure (Ko & Yao, 2006). The product was recrystallized from ethyl acetate  at room temperature to give block red crystals suitable for single-crystal X-ray diffraction.

### Refinement

In the refinement, the nitro group, C18 methyl group, acetyl group and some atoms of the dihydroquinolinone group (C9-C12/C16-C18/O3/O4) were modelled as disordered over two sites with the final site occupancies fixed at 0.57:0.43. Commands SADI and DFIX were used in the refinement to restrain some bond lengths. The relationship between the major and minor components of disorder is that of diastereomers.

All H atoms were visible in difference Fourier maps The N—H distance of H2A atom (for N4) was constrained to 0.86 (1) Å, while the displacement parameter of this atom was constrained with regard to its carrier atom: *U*_iso_(H4A) = 1.2*U*_eq_(N4). The remaining H atoms were placed in idealized positions, with C—H = 0.93, 0.98, 0.97 and 0.96 Å for aryl, methine, methylene and methyl groups, respectively, and *U*_iso_(H_aryl_/_methine_/_methylene_) = 1.2*U*_eq_(C_aryl/methine/methylene_) and *U*_iso_(H_methyl_) = 1.5*U*_eq_(C_methyl_).

### Crystal data

**Table:**

C_18_H_18_N_2_O_4_	*F*(000) = 688
*M**_r_* = 326.34	*D*_x_ = 1.353 Mg m^−^^3^
Monoclinic, *P*2_1_/*n*	Mo *K*α radiation, λ = 0.71073 Å
Hall symbol: -P 2yn	Cell parameters from 1160 reflections
*a* = 8.5368 (5) Å	θ = 2.2–19.3°
*b* = 17.0307 (6) Å	µ = 0.10 mm^−^^1^
*c* = 11.4759 (5) Å	*T* = 298 K
β = 106.143 (1)°	Block, yellow
*V* = 1602.67 (13) Å^3^	0.20 × 0.10 × 0.10 mm
*Z* = 4	

### Data collection

**Table:**

Bruker SMART APEX CCD area-detector diffractometer	3151 independent reflections
Radiation source: fine focus sealed Siemens Mo tube	1611 reflections with *I* > 2σ(*I*)
graphite	*R*_int_ = 0.094
0.3° wide ω exposures scans	θ_max_ = 26.0°, θ_min_ = 2.2°
Absorption correction: multi-scan (*SADABS*; Sheldrick, 1996)	*h* = −10→10
*T*_min_ = 0.971, *T*_max_ = 0.990	*k* = −21→14
9050 measured reflections	*l* = −13→14

### Refinement

**Table:**

Refinement on *F*^2^	Primary atom site location: structure-invariant direct methods
Least-squares matrix: full	Secondary atom site location: difference Fourier map
*R*[*F*^2^ > 2σ(*F*^2^)] = 0.061	Hydrogen site location: inferred from neighbouring sites
*wR*(*F*^2^) = 0.149	H atoms treated by a mixture of independent and constrained refinement
*S* = 0.90	*w* = 1/[σ^2^(*F*_o_^2^) + (0.0622*P*)^2^] where *P* = (*F*_o_^2^ + 2*F*_c_^2^)/3
3151 reflections	(Δ/σ)_max_ < 0.001
325 parameters	Δρ_max_ = 0.34 e Å^−^^3^
12 restraints	Δρ_min_ = −0.28 e Å^−^^3^

### Special details

**Table:**

Geometry. All esds (except the esd in the dihedral angle between two l.s. planes) are estimated using the full covariance matrix. The cell esds are taken into account individually in the estimation of esds in distances, angles and torsion angles; correlations between esds in cell parameters are only used when they are defined by crystal symmetry. An approximate (isotropic) treatment of cell esds is used for estimating esds involving l.s. planes.
Refinement. Refinement of F^2^ against ALL reflections. The weighted R-factor wR and goodness of fit S are based on F^2^, conventional R-factors R are based on F, with F set to zero for negative F^2^. The threshold expression of F^2^ > 2sigma(F^2^) is used only for calculating R-factors(gt) etc. and is not relevant to the choice of reflections for refinement. R-factors based on F^2^ are statistically about twice as large as those based on F, and R- factors based on ALL data will be even larger.

### Fractional atomic coordinates and isotropic or equivalent isotropic displacement parameters (Å^2^)

**Table:**

	*x*	*y*	*z*	*U*_iso_*/*U*_eq_	Occ. (<1)
C1	0.2737 (3)	0.20273 (14)	0.3479 (2)	0.0436 (6)	
C2	0.3036 (3)	0.18309 (15)	0.4692 (2)	0.0491 (7)	
H2	0.3974	0.2011	0.5255	0.059*	
C3	0.1941 (4)	0.13684 (16)	0.5063 (2)	0.0543 (7)	
C4	0.0532 (4)	0.10958 (16)	0.4267 (3)	0.0615 (8)	
H4	−0.0197	0.0784	0.4532	0.074*	
C5	0.0234 (3)	0.12982 (17)	0.3068 (3)	0.0621 (8)	
H5	−0.0714	0.1123	0.2512	0.075*	
C6	0.1317 (3)	0.17567 (16)	0.2676 (2)	0.0526 (7)	
H6	0.1089	0.1886	0.1858	0.063*	
C7	0.3954 (3)	0.25236 (15)	0.3038 (2)	0.0455 (7)	
H7	0.4970	0.2547	0.3697	0.055*	
C8	0.4336 (3)	0.21449 (16)	0.1960 (2)	0.0474 (7)	
C9	0.545 (5)	0.151 (2)	0.220 (2)	0.053 (9)	0.57
C10	0.5914 (19)	0.1135 (11)	0.1123 (15)	0.075 (5)	0.57
H10A	0.6193	0.0587	0.1290	0.090*	0.57
H10B	0.6843	0.1402	0.0974	0.090*	0.57
C11	0.4385 (8)	0.1214 (3)	−0.0005 (4)	0.0700 (17)	0.57
H11A	0.4659	0.0995	−0.0704	0.084*	0.57
H11B	0.3515	0.0896	0.0139	0.084*	0.57
C12	0.3717 (10)	0.2063 (7)	−0.0331 (9)	0.039 (2)	0.57
H12A	0.4451	0.2365	−0.0668	0.047*	0.57
H12B	0.2651	0.2049	−0.0918	0.047*	0.57
O3	0.6237 (12)	0.1317 (7)	0.3215 (8)	0.044 (2)	0.57
C16	0.356 (2)	0.3899 (17)	0.3823 (18)	0.052 (7)	0.57
C17	0.2873 (9)	0.4703 (5)	0.3779 (6)	0.0517 (18)	0.57
H17A	0.3114	0.4913	0.4586	0.077*	0.57
H17B	0.1712	0.4684	0.3432	0.077*	0.57
H17C	0.3349	0.5033	0.3290	0.077*	0.57
C18	0.1732 (18)	0.4344 (7)	0.1111 (9)	0.050 (3)	0.57
H18A	0.0921	0.4436	0.1532	0.076*	0.57
H18B	0.1210	0.4288	0.0259	0.076*	0.57
H18C	0.2473	0.4780	0.1240	0.076*	0.57
O4	0.439 (3)	0.3647 (9)	0.4817 (13)	0.103 (6)	0.57
C9'	0.368 (3)	0.385 (2)	0.378 (2)	0.075 (14)	0.43
C10'	0.3390 (15)	0.4734 (9)	0.3478 (11)	0.085 (4)	0.43
H10C	0.4315	0.4959	0.3262	0.102*	0.43
H10D	0.3224	0.5019	0.4165	0.102*	0.43
C11'	0.1799 (10)	0.4766 (5)	0.2359 (8)	0.089 (3)	0.43
H11C	0.0919	0.4480	0.2550	0.107*	0.43
H11D	0.1456	0.5307	0.2184	0.107*	0.43
C12'	0.220 (3)	0.4386 (9)	0.1223 (14)	0.067 (6)	0.43
H12C	0.1249	0.4391	0.0524	0.081*	0.43
H12D	0.3080	0.4665	0.1022	0.081*	0.43
O3'	0.414 (3)	0.3620 (10)	0.4820 (15)	0.061 (4)	0.43
C16'	0.549 (7)	0.146 (2)	0.219 (3)	0.047 (12)	0.43
C17'	0.566 (2)	0.0938 (13)	0.1187 (17)	0.047 (4)	0.43
H17D	0.6219	0.1213	0.0696	0.071*	0.43
H17E	0.4592	0.0788	0.0698	0.071*	0.43
H17F	0.6261	0.0477	0.1522	0.071*	0.43
C18'	0.379 (2)	0.1953 (11)	−0.0320 (13)	0.105 (8)	0.43
H18D	0.4920	0.1827	−0.0213	0.158*	0.43
H18E	0.3405	0.2274	−0.1030	0.158*	0.43
H18F	0.3166	0.1477	−0.0416	0.158*	0.43
O4'	0.608 (2)	0.1250 (13)	0.3267 (16)	0.103 (6)	0.43
C13	0.3612 (3)	0.24079 (15)	0.0812 (2)	0.0454 (7)	
C14	0.2679 (3)	0.35884 (16)	0.1597 (2)	0.0505 (7)	
C15	0.3347 (3)	0.33563 (17)	0.2754 (2)	0.0517 (7)	
N1	0.2306 (5)	0.1151 (2)	0.6349 (3)	0.0779 (9)	
N2	0.2681 (3)	0.30700 (13)	0.06613 (18)	0.0510 (6)	
H2A	0.227 (3)	0.3210 (14)	−0.0089 (11)	0.061*	
O1	0.370 (2)	0.1286 (8)	0.7031 (14)	0.092 (3)	0.57
O2	0.123 (4)	0.0762 (16)	0.668 (3)	0.105 (6)	0.57
O1'	0.326 (3)	0.1589 (11)	0.7037 (19)	0.098 (4)	0.43
O2'	0.159 (5)	0.0643 (18)	0.665 (3)	0.098 (6)	0.43

### Atomic displacement parameters (Å^2^)

**Table:**

	*U*^11^	*U*^22^	*U*^33^	*U*^12^	*U*^13^	*U*^23^
C1	0.0465 (16)	0.0500 (16)	0.0340 (14)	0.0065 (13)	0.0109 (12)	−0.0020 (12)
C2	0.0530 (17)	0.0575 (18)	0.0384 (15)	0.0020 (13)	0.0151 (13)	−0.0041 (12)
C3	0.065 (2)	0.0602 (19)	0.0450 (17)	0.0081 (15)	0.0284 (15)	0.0028 (14)
C4	0.062 (2)	0.0566 (19)	0.075 (2)	0.0036 (15)	0.0347 (17)	−0.0002 (16)
C5	0.0481 (18)	0.074 (2)	0.061 (2)	−0.0052 (15)	0.0105 (15)	−0.0074 (16)
C6	0.0460 (17)	0.0666 (19)	0.0423 (16)	−0.0009 (14)	0.0072 (13)	0.0006 (13)
C7	0.0422 (15)	0.0610 (19)	0.0303 (14)	−0.0039 (13)	0.0053 (11)	−0.0003 (12)
C8	0.0433 (16)	0.0598 (19)	0.0401 (16)	−0.0037 (14)	0.0134 (12)	−0.0001 (13)
C9	0.045 (16)	0.072 (13)	0.039 (17)	−0.001 (11)	0.008 (12)	−0.009 (10)
C10	0.085 (8)	0.081 (10)	0.064 (6)	−0.013 (5)	0.031 (6)	−0.009 (6)
C11	0.107 (5)	0.068 (4)	0.030 (3)	0.019 (3)	0.011 (3)	−0.006 (3)
C12	0.039 (4)	0.043 (5)	0.039 (6)	−0.006 (3)	0.018 (4)	−0.004 (4)
O3	0.043 (3)	0.058 (4)	0.024 (4)	0.010 (3)	0.000 (3)	−0.010 (3)
C16	0.058 (9)	0.055 (12)	0.042 (13)	−0.007 (8)	0.014 (9)	−0.003 (9)
C17	0.058 (4)	0.055 (4)	0.042 (4)	−0.007 (4)	0.014 (3)	−0.003 (3)
C18	0.054 (9)	0.052 (5)	0.047 (4)	−0.007 (4)	0.016 (4)	0.0003 (3)
O4	0.145 (11)	0.097 (8)	0.048 (8)	0.009 (6)	−0.007 (6)	0.007 (5)
C9'	0.10 (2)	0.059 (19)	0.06 (2)	−0.018 (16)	0.015 (17)	−0.007 (14)
C10'	0.117 (10)	0.078 (7)	0.090 (8)	−0.001 (7)	0.078 (7)	0.007 (5)
C11'	0.088 (6)	0.067 (6)	0.113 (7)	0.018 (5)	0.031 (6)	−0.001 (5)
C12'	0.032 (8)	0.058 (8)	0.102 (10)	−0.009 (5)	0.003 (6)	−0.003 (6)
O3'	0.103 (7)	0.045 (7)	0.037 (9)	−0.001 (5)	0.023 (7)	−0.023 (6)
C16'	0.04 (2)	0.060 (16)	0.04 (2)	−0.004 (13)	0.013 (17)	0.000 (13)
C17'	0.043 (6)	0.060 (9)	0.040 (6)	−0.004 (6)	0.013 (5)	0.000 (5)
C18'	0.200 (16)	0.076 (13)	0.038 (10)	0.021 (11)	0.031 (9)	0.005 (8)
O4'	0.127 (12)	0.105 (11)	0.074 (10)	0.036 (9)	0.025 (8)	0.020 (7)
C13	0.0477 (16)	0.0515 (17)	0.0376 (15)	−0.0011 (13)	0.0132 (12)	0.0012 (12)
C14	0.0536 (18)	0.0520 (19)	0.0470 (17)	−0.0070 (14)	0.0160 (14)	−0.0003 (14)
C15	0.0582 (18)	0.0552 (19)	0.0418 (17)	−0.0071 (14)	0.0143 (14)	−0.0034 (14)
N1	0.089 (3)	0.091 (3)	0.063 (2)	0.003 (2)	0.0351 (19)	0.0093 (19)
N2	0.0605 (15)	0.0587 (15)	0.0303 (12)	0.0057 (12)	0.0068 (11)	0.0025 (11)
O1	0.104 (7)	0.126 (10)	0.046 (3)	0.004 (5)	0.021 (4)	0.016 (5)
O2	0.101 (9)	0.130 (9)	0.102 (7)	0.017 (5)	0.059 (5)	0.030 (5)
O1'	0.125 (13)	0.115 (12)	0.052 (5)	−0.003 (7)	0.023 (7)	−0.003 (7)
O2'	0.117 (17)	0.104 (11)	0.090 (8)	−0.015 (12)	0.058 (9)	0.044 (8)

### Geometric parameters (Å, °)

**Table:**

C1—C6	1.383 (3)	C17—H17C	0.9600
C1—C2	1.384 (3)	C18—C14	1.540 (12)
C1—C7	1.530 (3)	C18—H18A	0.9600
C2—C3	1.378 (4)	C18—H18B	0.9600
C2—H2	0.9300	C18—H18C	0.9600
C3—C4	1.374 (4)	C9'—O3'	1.21 (3)
C3—N1	1.468 (4)	C9'—C15	1.41 (3)
C4—C5	1.372 (4)	C9'—C10'	1.54 (4)
C4—H4	0.9300	C10'—C11'	1.590 (13)
C5—C6	1.378 (4)	C10'—H10C	0.9700
C5—H5	0.9300	C10'—H10D	0.9700
C6—H6	0.9300	C11'—C12'	1.575 (14)
C7—C8	1.509 (3)	C11'—H11C	0.9700
C7—C15	1.514 (4)	C11'—H11D	0.9700
C7—H7	0.9800	C12'—C14	1.449 (14)
C8—C13	1.365 (3)	C12'—H12C	0.9700
C8—C9	1.41 (3)	C12'—H12D	0.9700
C8—C16'	1.51 (3)	C16'—O4'	1.25 (3)
C9—O3	1.22 (2)	C16'—C17'	1.49 (3)
C9—C10	1.54 (3)	C17'—H17D	0.9600
C10—C11	1.567 (14)	C17'—H17E	0.9600
C10—H10A	0.9700	C17'—H17F	0.9600
C10—H10B	0.9700	C18'—C13	1.558 (14)
C11—C12	1.562 (12)	C18'—H18D	0.9600
C11—H11A	0.9700	C18'—H18E	0.9600
C11—H11B	0.9700	C18'—H18F	0.9600
C12—C13	1.463 (10)	C13—N2	1.362 (3)
C12—H12A	0.9700	C14—C15	1.350 (3)
C12—H12B	0.9700	C14—N2	1.391 (3)
C16—O4	1.24 (2)	N1—O2'	1.17 (3)
C16—C17	1.48 (3)	N1—O1'	1.22 (3)
C16—C15	1.51 (3)	N1—O1	1.251 (19)
C17—H17A	0.9600	N1—O2	1.28 (2)
C17—H17B	0.9600	N2—H2A	0.868 (10)
			
C6—C1—C2	118.2 (2)	C9'—C10'—C11'	105.1 (13)
C6—C1—C7	120.9 (2)	C9'—C10'—H10C	110.7
C2—C1—C7	120.9 (2)	C11'—C10'—H10C	110.7
C3—C2—C1	119.9 (2)	C9'—C10'—H10D	110.7
C3—C2—H2	120.1	C11'—C10'—H10D	110.7
C1—C2—H2	120.1	H10C—C10'—H10D	108.8
C4—C3—C2	122.1 (3)	C12'—C11'—C10'	108.7 (10)
C4—C3—N1	119.0 (3)	C12'—C11'—H11C	110.0
C2—C3—N1	118.8 (3)	C10'—C11'—H11C	110.0
C5—C4—C3	117.8 (3)	C12'—C11'—H11D	110.0
C5—C4—H4	121.1	C10'—C11'—H11D	110.0
C3—C4—H4	121.1	H11C—C11'—H11D	108.3
C4—C5—C6	121.1 (3)	C14—C12'—C11'	104.4 (9)
C4—C5—H5	119.5	C14—C12'—H12C	110.9
C6—C5—H5	119.5	C11'—C12'—H12C	110.9
C5—C6—C1	121.0 (3)	C14—C12'—H12D	110.9
C5—C6—H6	119.5	C11'—C12'—H12D	110.9
C1—C6—H6	119.5	H12C—C12'—H12D	108.9
C8—C7—C15	111.3 (2)	O4'—C16'—C17'	120 (3)
C8—C7—C1	110.9 (2)	O4'—C16'—C8	117 (3)
C15—C7—C1	111.4 (2)	C17'—C16'—C8	122 (2)
C8—C7—H7	107.7	C16'—C17'—H17D	109.5
C15—C7—H7	107.7	C16'—C17'—H17E	109.5
C1—C7—H7	107.7	H17D—C17'—H17E	109.5
C13—C8—C9	122.5 (12)	C16'—C17'—H17F	109.5
C13—C8—C16'	121.4 (13)	H17D—C17'—H17F	109.5
C13—C8—C7	120.5 (3)	H17E—C17'—H17F	109.5
C9—C8—C7	117.0 (12)	C13—C18'—H18D	109.5
C16'—C8—C7	118.0 (13)	C13—C18'—H18E	109.5
O3—C9—C8	124 (2)	H18D—C18'—H18E	109.5
O3—C9—C10	117 (2)	C13—C18'—H18F	109.5
C8—C9—C10	118.2 (17)	H18D—C18'—H18F	109.5
C9—C10—C11	106.6 (18)	H18E—C18'—H18F	109.5
C9—C10—H10A	110.4	N2—C13—C8	119.1 (2)
C11—C10—H10A	110.4	N2—C13—C12	113.4 (5)
C9—C10—H10B	110.4	C8—C13—C12	127.5 (5)
C11—C10—H10B	110.4	N2—C13—C18'	119.7 (7)
H10A—C10—H10B	108.6	C8—C13—C18'	121.2 (7)
C12—C11—C10	116.2 (9)	C15—C14—N2	118.9 (3)
C12—C11—H11A	108.2	C15—C14—C12'	125.0 (7)
C10—C11—H11A	108.2	N2—C14—C12'	115.3 (7)
C12—C11—H11B	108.2	C15—C14—C18	129.5 (4)
C10—C11—H11B	108.2	N2—C14—C18	111.4 (4)
H11A—C11—H11B	107.4	C14—C15—C9'	125.2 (13)
C13—C12—C11	105.4 (6)	C14—C15—C16	122.9 (9)
C13—C12—H12A	110.7	C14—C15—C7	120.8 (2)
C11—C12—H12A	110.7	C9'—C15—C7	113.9 (13)
C13—C12—H12B	110.7	C16—C15—C7	116.3 (9)
C11—C12—H12B	110.7	O2'—N1—O1'	125 (2)
H12A—C12—H12B	108.8	O2'—N1—O1	116 (2)
O4—C16—C17	118 (2)	O1'—N1—O2	122.1 (19)
O4—C16—C15	116 (2)	O1—N1—O2	123.1 (17)
C17—C16—C15	125.6 (13)	O1'—N1—C3	114.4 (11)
C14—C18—H18A	109.5	O1—N1—C3	118.8 (8)
C14—C18—H18B	109.5	O2—N1—C3	117.7 (16)
C14—C18—H18C	109.5	C13—N2—C14	123.6 (2)
O3'—C9'—C15	124 (3)	C13—N2—H2A	114.5 (17)
O3'—C9'—C10'	122 (3)	C14—N2—H2A	120.3 (17)
C15—C9'—C10'	114.3 (19)		
			
C6—C1—C2—C3	0.9 (4)	C11—C12—C13—C8	16.2 (7)
C7—C1—C2—C3	−178.5 (2)	C11'—C12'—C14—C15	−27.2 (16)
C1—C2—C3—C4	−0.7 (4)	C11'—C12'—C14—N2	163.7 (8)
C1—C2—C3—N1	178.4 (3)	N2—C14—C15—C9'	169.0 (11)
C2—C3—C4—C5	0.1 (4)	C12'—C14—C15—C9'	0.3 (15)
N1—C3—C4—C5	−179.0 (3)	C18—C14—C15—C9'	−17.2 (13)
C3—C4—C5—C6	0.3 (4)	N2—C14—C15—C16	173.7 (7)
C4—C5—C6—C1	0.0 (4)	C12'—C14—C15—C16	5.0 (13)
C2—C1—C6—C5	−0.6 (4)	C18—C14—C15—C16	−12.5 (11)
C7—C1—C6—C5	178.8 (2)	N2—C14—C15—C7	−5.3 (4)
C6—C1—C7—C8	−49.3 (3)	C12'—C14—C15—C7	−174.1 (10)
C2—C1—C7—C8	130.1 (2)	C18—C14—C15—C7	168.5 (7)
C6—C1—C7—C15	75.2 (3)	O3'—C9'—C15—C14	173.2 (14)
C2—C1—C7—C15	−105.4 (3)	C10'—C9'—C15—C14	−6.7 (19)
C15—C7—C8—C13	−23.7 (3)	C10'—C9'—C15—C7	168.0 (10)
C1—C7—C8—C13	100.9 (3)	O4—C16—C15—C14	−170.3 (11)
C15—C7—C8—C9	157 (2)	C17—C16—C15—C14	9.1 (16)
C1—C7—C8—C9	−79 (2)	O4—C16—C15—C7	8.8 (15)
C15—C7—C8—C16'	158 (3)	C17—C16—C15—C7	−171.9 (9)
C1—C7—C8—C16'	−77 (3)	C8—C7—C15—C14	22.7 (3)
C13—C8—C9—O3	172 (3)	C1—C7—C15—C14	−101.6 (3)
C16'—C8—C9—O3	−137 (100)	C8—C7—C15—C9'	−152.2 (10)
C7—C8—C9—O3	−8(5)	C1—C7—C15—C9'	83.5 (10)
C13—C8—C9—C10	3(5)	C8—C7—C15—C16	−156.4 (7)
C16'—C8—C9—C10	53 (100)	C1—C7—C15—C16	79.3 (7)
C7—C8—C9—C10	−178 (2)	C2—C3—N1—O2'	−165 (2)
O3—C9—C10—C11	158 (3)	C4—C3—N1—O1'	−158.8 (8)
C8—C9—C10—C11	−31 (4)	C2—C3—N1—O1'	22.1 (9)
C9—C10—C11—C12	56 (2)	C4—C3—N1—O1	168.2 (7)
C10—C11—C12—C13	−48.0 (10)	C2—C3—N1—O1	−10.9 (8)
O3'—C9'—C10'—C11'	−141.3 (17)	C4—C3—N1—O2	−5.0 (15)
C15—C9'—C10'—C11'	38.6 (16)	C2—C3—N1—O2	175.9 (15)
C9'—C10'—C11'—C12'	−67.2 (13)	C8—C13—N2—C14	13.6 (4)
C10'—C11'—C12'—C14	60.0 (14)	C12—C13—N2—C14	−165.8 (4)
C7—C8—C13—N2	7.2 (4)	C18'—C13—N2—C14	−168.1 (8)
C7—C8—C13—C12	−173.5 (5)	C15—C14—N2—C13	−14.6 (4)
C7—C8—C13—C18'	−171.1 (8)	C12'—C14—N2—C13	155.3 (9)
C11—C12—C13—N2	−164.5 (4)	C18—C14—N2—C13	170.6 (6)

### Hydrogen-bond geometry (Å, °)

**Table:**

*D*—H···*A*	*D*—H	H···*A*	*D*···*A*	*D*—H···*A*
N2—H2A···O3^i^	0.87 (1)	2.06 (1)	2.927 (8)	173 (2)
C6—H6···O4^i^	0.93	2.56	3.309 (16)	138
C18—H18A···O1^i^	0.96	2.46	3.239 (18)	138
C18—H18B···O3^i^	0.96	2.57	3.422 (15)	148
C11—H11A···O1^ii^	0.97	2.55	3.290 (16)	133
C12—H12A···Cg1^iii^	0.97	2.76	3.71 (1)	166

Symmetry codes: (i) *x*−1/2, −*y*+1/2, *z*−1/2; (ii) *x*, *y*, *z*−1; (iii) *x*+1/2, −*y*+1/2, *z*−1/2.
